# The PET-derived tumor-to-blood standard uptake ratio (SUR) is superior to tumor SUV as a surrogate parameter of the metabolic rate of FDG

**DOI:** 10.1186/2191-219X-3-77

**Published:** 2013-11-23

**Authors:** Jörg van den Hoff, Liane Oehme, Georg Schramm, Jens Maus, Alexandr Lougovski, Jan Petr, Bettina Beuthien-Baumann, Frank Hofheinz

**Affiliations:** 1 PET Center, Institute of Radiopharmaceutical Cancer Research, Helmholtz-Zentrum Dresden-Rossendorf, Dresden 01328, Germany; 2 Department of Nuclear Medicine, University Hospital Carl Gustav Carus, Technische Universität Dresden, Dresden 01307, Germany

**Keywords:** SUV, Tumor-to-blood ratio, PET, PET/CT, Therapy response control, FDG

## Abstract

**Background:**

The standard uptake value (SUV) approach in oncological positron emission tomography has known shortcomings, all of which affect the reliability of the SUV as a surrogate of the targeted quantity, the metabolic rate of [^18^F]fluorodeoxyglucose (FDG), *K*_*m*_. Among the shortcomings are time dependence, susceptibility to errors in scanner and dose calibration, insufficient correlation between systemic distribution volume and body weight, and, consequentially, residual inter-study variability of the arterial input function (AIF) despite SUV normalization. Especially the latter turns out to be a crucial factor adversely affecting the correlation between SUV and *K*_*m*_ and causing inter-study variations of tumor SUVs that do not reflect actual changes of the metabolic uptake rate. In this work, we propose to replace tumor SUV by the tumor-to-blood standard uptake ratio (SUR) in order to distinctly improve the linear correlation with *K*_*m*_.

**Methods:**

Assuming irreversible FDG kinetics, SUR can be expected to exhibit a much better linear correlation to *K*_*m*_ than SUV. The theoretical derivation for this prediction is given and evaluated in a group of nine patients with liver metastases of colorectal cancer for which 15 fully dynamic investigations were available and *K*_*m*_ could thus be derived from conventional Patlak analysis.

**Results:**

For any fixed time point *T* at sufficiently late times post injection, the Patlak equation predicts a linear correlation between SUR and *K*_*m*_ under the following assumptions: (1) approximate shape invariance (but arbitrary scale) of the AIF across scans/patients and (2) low variability of the apparent distribution volume *V*_*r*_ (the intercept of the Patlak Plot). This prediction - and validity of the underlying assumptions - has been verified in the investigated patient group. Replacing tumor SUVs by SURs does improve the linear correlation of the respective parameter with *K*_*m*_ from *r* = 0.61 to *r* = 0.98.

**Conclusions:**

SUR is an easily measurable parameter that is highly correlated to *K*_*m*_. In this respect, it is clearly superior to SUV. Therefore, SUR should be seriously considered as a drop-in replacement for SUV-based approaches.

## Background

Today, in the clinical oncological setting, the standard uptake value (SUV, g/ml), defined as the tracer concentration at a certain time point normalized to injected dose per unit body weight is essentially the only means for quantitative evaluation of [^18^F]fluorodeoxyglucose (FDG) positron emission tomography (PET). While it is perfectly possible to quantify the actually targeted parameter, namely the absolute metabolic rate of glucose consumption (or, rather, the rate of irreversible FDG accumulation, *K*_*m*_), the conventional approaches such as the Patlak plot [1,2] require to a varying degree dynamic imaging and also determination of the full arterial input function both of which requirements are not compatible with oncological whole body imaging. For this reason, different alternative approaches have been investigated in the past which have tried to facilitate the quantification of *K*_*m*_ in order to make it more suitable for the oncological setting [3-8]. However, none of these approaches have so far gained widespread acceptance since they either rest on quite restrictive assumptions and approximations or still impose demands on data acquisition and evaluation that are significantly higher than for SUV-based approaches. Recently, we have shown that a purely image-based quantification of *K*_*m*_ might be performed that only requires a dual time point PET measurement [9]. This seems to constitute the minimum requirement necessary to actually determine this parameter. While such dual time point protocols could be considered acceptable for clinical routine, they still do somewhat increase complexity of the work flow and data evaluation and will probably only augment but not replace static oncological imaging.

The SUV approach, on the other hand, has several known shortcomings [10-12] all of which affect the reliability of the SUV as a surrogate of *K*_*m*_ (and the metabolic rate of glucose consumption). The following are among these shortcomings: 

● Time dependence of the SUV (requiring to strictly standardize the time point after injection chosen for imaging and evaluation),

● Susceptibility to errors in scanner calibration,

● Insufficient correlation between systemic distribution volume and body weight (leading to variants of the SUV approach using lean body mass (SUV_lbm_) [13] or body surface area (SUV_bsa_) [14] for normalization), and, consequentially, residual inter-study variability of the arterial input function (AIF) despite SUV normalization.

Especially the latter point turns out to be a crucial factor adversely affecting the correlation between SUV and *K*_*m*_ and, more generally, leading to uncontrolled inter-study variability of tumor SUVs.

In this work, we propose to replace tumor SUVs by the tumor-to-blood uptake ratio, called standard uptake ratio (SUR), in the following: SUR can be expected on theoretical grounds to exhibit a much better linear correlation to *K*_*m*_ than SUV. The derivation for this prediction is given and evaluated in a group of nine patients with liver metastases of colorectal cancer for which 15 fully dynamical investigations were available and *K*_*m*_ could thus be derived from conventional Patlak analysis.

## Methods

### Theory

We start with the standard Patlak formula:

(1) $$\frac{c_{t}(t)}{c_{a}(t)}=K_{m}\times \frac{\int_{0}^{t}c_{a}(s)\text{ds}}{c_{a}(t)}+V_{r}$$

where *K*_*m*_ is the metabolic trapping rate and *V*_*r*_ is the so-called apparent volume of distribution. This equation is valid for times later than some time point *t*^∗^ (typically *t*^∗^≈15 to 20 min post injection (p.i.)).

This equation is of course valid independent of the units chosen to specify the tracer concentrations, but in the following we view *c*_*t*_(*t*), *c*_*a*_(*t*) as being expressed in SUV units.

For the sake of argument, we further assume the following: 

1. All input functions exhibit a common shape *b*_*a*_(*t*), i.e., are approximately shape invariant across all considered investigations and only differ by an investigation-specific scaling factor *N*: *c*_*a*_(*t*)≈*N*×*b*_*a*_(*t*) where *N* is measured in the same units as *c*_*a*_ and the shape function *b*_*a*_(*t*) is dimensionless. Taking into account that all SUV-based evaluations require that the measurement time is strictly standardized and denoting this fixed time point as *T*, we choose the arbitrary normalization of *b*_*a*_(*t*) in such a way that *b*_*a*_(*T*)=1. The scaling factor *N* then becomes identical to the blood concentration at the chosen time point: *N*=*c*_*a*_(*T*).

2. Variability of the apparent volume of distribution, *V*_*r*_, is small for the considered tumor entity across all considered investigations, and it can thus be taken to be approximately constant:

$$V_{r}\approx {\bar{V}}_{r}=\text{const.}$$

It then follows immediately from Equation 1 that the tumor-to-blood uptake ratio at time *t*=*T* is related to the metabolic rate *K*_*m*_ according to

(2) $$\frac{c_{t}(T)}{c_{a}(T)}=\text{SUR}(T)\approx \Theta (T)\times K_{m}+{\bar{V}}_{r}$$

where

(3) $$\begin{array}{l} \Theta (T)=\frac{\text{AUC}(T)}{c_{a}(T)}=\frac{\int_{0}^{T}c_{a}(s)\text{ds}}{c_{a}(T)}\\ \;=\frac{\int_{0}^{T}c_{a}(T)\;b_{a}(s)\;\text{ds}}{c_{a}(T)}=\int_{0}^{T}b_{a}(s)\text{ds}\;. \end{array}$$

Obviously, *Θ* is identical to the independent (*x*-) coordinate of the Patlak plot which dimensionally is a time (sometimes called ‘Patlak’s funny time’). The crucial point here is to realize that *Θ* is not influenced by scale changes of *c*_*a*_(*t*). If *b*_*a*_(*t*) can be assumed to have a fixed shape (at least for ‘most of the time’: shape variations during the bolus passage do not sizably influence the integral up to the late time point *T*), *Θ*(*T*) is in fact constant across all considered investigations for any given choice of the measurement time point *T*. The above equations show that if the used assumptions are adequate, one can expect a linear relation between SUR and *K*_*m*_ (in order to improve readability, any dependence on *T* is no longer stated explicitly here and in the following but has to be kept in mind).

Contrary to SUR, the tissue SUV itself (i.e., *c*_*t*_) would only be related linearly to *K*_*m*_ if the input functions - when expressed in SUV units - were in fact completely identical instead of only shape invariant across different investigations and target structures. This is directly obvious from Equation 2 when multiplying both sides by *c*_*a*_:

(4) $$c_{t}=\text{SUV}\approx \text{AUC}\times K_{m}+{\bar{V}}_{r}\times c_{a}$$

since now, both *c*_*a*_ and the area under the input curve would be required to assume investigation-independent fixed values. This trivially is the case for intra-scan comparisons of different target structures but not at all for inter-subject comparisons (or inter-scan comparisons of the same patient). Actually, *c*_*a*_ is known to exhibit substantial variability across scans even when expressed in SUV units [13].

To summarize, the difference between SUV and SUR can be stated as follows : SUR is linearly related to *K*_*m*_ under a distinctly weaker assumption (common shape of input function required) than SUV (identical input function required).

The prediction following from the above considerations is very simple: SUR should be a distinctly better surrogate parameter of *K*_*m*_ than the tissue SUV since it is expected to exhibit a much higher linear correlation to *K*_*m*_. We have tested this prediction in the present study focusing on the potentially most relevant case of investigations in the trunk where *c*_*a*_ can be easily derived within the reconstructed image volumes from a suitable region in either the aorta or the left ventricle of the heart.

### Study sample

Nine male patients with liver metastases of colorectal cancer were included retrospectively (age, 48 to 76 years (mean 62.8); weight, 73 to 100 kg (mean 85.5)). For each patient, one to three dynamic PET scans of 60 min in duration were performed (altogether 15 scans). Scans started immediately after injection of 346 to 430 MBq FDG administrated as bolus over 10 to 20 s. Diabetics were excluded, and no forced diuresis was used. The scans were conducted with an ECAT EXACT HR^+^ (Siemens/CTI, Knoxville, TN, USA). The acquired data were sorted into 23 to 31 frames with 10 to 20 s in duration during bolus passage, 30 to 150 s in duration until 10 min p.i., and 300 s in duration afterwards. Tomographic images were reconstructed using attenuation weighted OSEM reconstruction (six iterations, 16 subsets, 6-mm FWHM Gaussian filter).

The study protocol has been approved by the local Clinical Institutional Review Board and has complied with the Declaration of Helsinki.

### Data evaluation

The AIF was determined from a roughly cylindrical three-dimensional (3D) region of interest (ROI) centered in the aorta. To exclude partial volume effects, a concentric safety margin of at least 1 cm was used in the transaxial plane. To compensate for the resulting small transaxial diameter, the ROIs were extended axially (along the aorta) for about 10 cm. This resulted in sufficiently large ROI volumes (mean ± standard deviation 7.3±1.8 cm^3^) and ensured sufficiently high statistical accuracy of the derived AIF values.

Three-dimensional ROIs were defined in 22 lesions, and the respective tissue response functions were computed. ROI definition was performed using the ROVER software (ABX, Radeberg, Germany; [15,16]). Further data analysis was carried out using the R software for statistical computing [17].

For all 22 lesions, *K*_*m*_ was derived from conventional Patlak analysis of the full dynamic data later than 20 min p.i. (at which time, all Patlak plots were already linear). The summed late time data between 50 and 60 min p.i. (corresponding to *T*=55 min) were used to determine *c*_*a*_ and *c*_*t*_. For each ROI mean values of SUV (= *c*_*t*_), SUR (= *c*_*t*_/ *c*_*a*_) and *K*_*m*_ were computed. Linear regression analysis was performed for SUV vs. *K*_*m*_ and SUR vs. *K*_*m*_, respectively.

The inter-study variability of *c*_*a*_ was determined in SUV units (SUV^*a*^), SUV_lbm_ units (${\text{SUV}}_{\text{lbm}}^{a}$), and SUV_bsa_ units (${\text{SUV}}_{\text{bsa}}^{a}$), respectively. For better comparability, ${\text{SUV}}_{\text{bsa}}^{a}$ was normalized such that the mean ${\text{SUV}}_{\text{bsa}}^{a}$ equals the mean ${\text{SUV}}_{\text{lbm}}^{a}$ as described in [18]. Additionally, the variability of area under the curve (AUC) and of *Θ*= AUC/ *c*_*a*_ was investigated.

## Results and discussion

### Results

**Figure 1:**
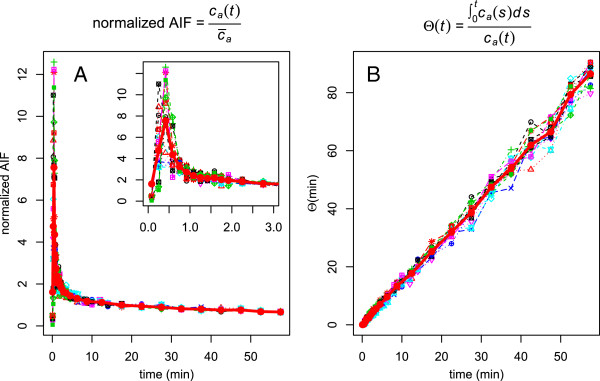
**Inter-study variability of AIF shape and *Θ *(*t*) = AUC (*t*)/*c***_***a***_**(*t*) in the available 15 data sets.** The thick red lines represent the group-averaged curves. **(A)** AIF normalized to the mean value (time-average) of the respective curve. As can be seen, the shape of all AIF curves is very similar beyond *t*≈1 min. **(B)***Θ*(*t*), the ratio of the AUC up to time *t* divided by *c*_*a*_(*t*), exhibits only small inter-study variability. Averaging the last two points of these curves (acquisition time from 50 to 60 min p.i.) yields the data presented in more detail below.

Figure 1 shows the complete time course of, both the AIF - normalized to the time-average of the respective curve, ${\bar{c}}_{a}=\frac{1}{T}\int_{0}^{T}c_{a}(s)\text{ds}$ - and *Θ*(*t*). As can be seen, the normalized AIFs exhibit only a very small variability beyond *t*≈1 min, demonstrating that the assumption of approximate shape invariance is valid. Consequently, inter-study variability of *Θ*(*t*) is small, too. This is especially true at late times which are the relevant ones in the present context.

**Figure 2:**
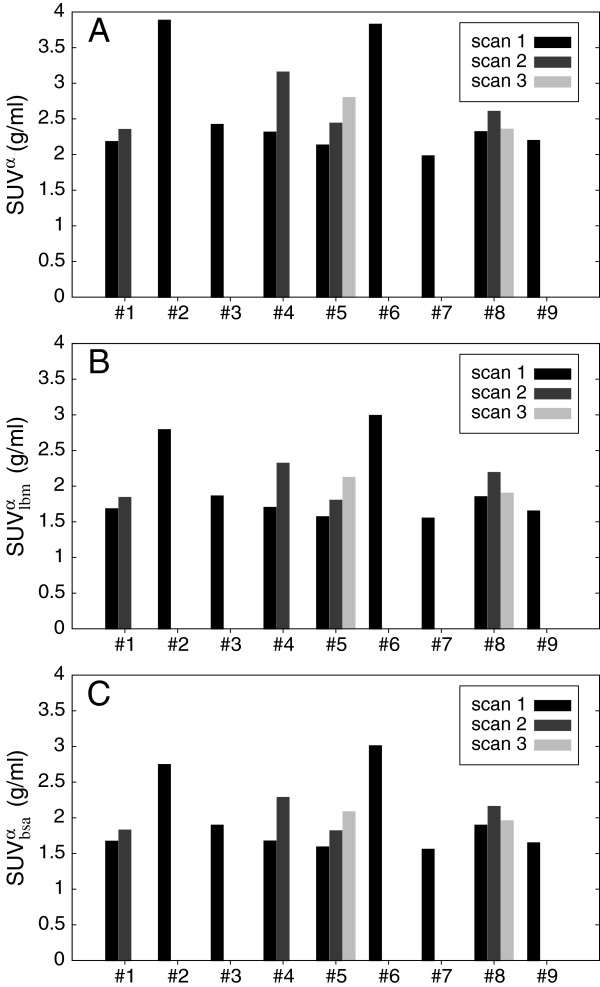
**Inter-study variability of *c***_***a***_** (55 min p.i.) in nine subjects and 15 scans expressed in three SUV units.(A)** Conventional SUV (body weight normalized), **(B)** SUV_lbm_ (lean body mass normalized), and **(C)** SUV_bsa_ (body surface area normalized).

In Figure 2, the inter-study variability of *c*_*a*_ is investigated in detail for the chosen time point *T*=55 min. The degree of variability is not reduced when using either SUV_lbm_ (Figure 2B) or SUV_bsa_ (Figure 2C) instead of conventional (body weight normalized) SUV (Figure 2A): on average, we obtain *c*_*a*_=2.59±0.58 (SUV), 1.99±0.43 (SUV_lbm_), and 1.99±0.42 (SUV_bsa_), respectively, corresponding to a standard deviation of 22% to 23% relative to the respective mean. Moreover, in our patient group, *c*_*a*_ and serum glucose concentration (range 4.8 to 10.3 µmol/ml) were found to be essentially uncorrelated (*r*=−0.14,*P*<0.65). With the possible exception of subjects 2 and 6, it can also be observed that the intra-subject variability is of the same order of magnitude than the inter-subject variability.

**Figure 3:**
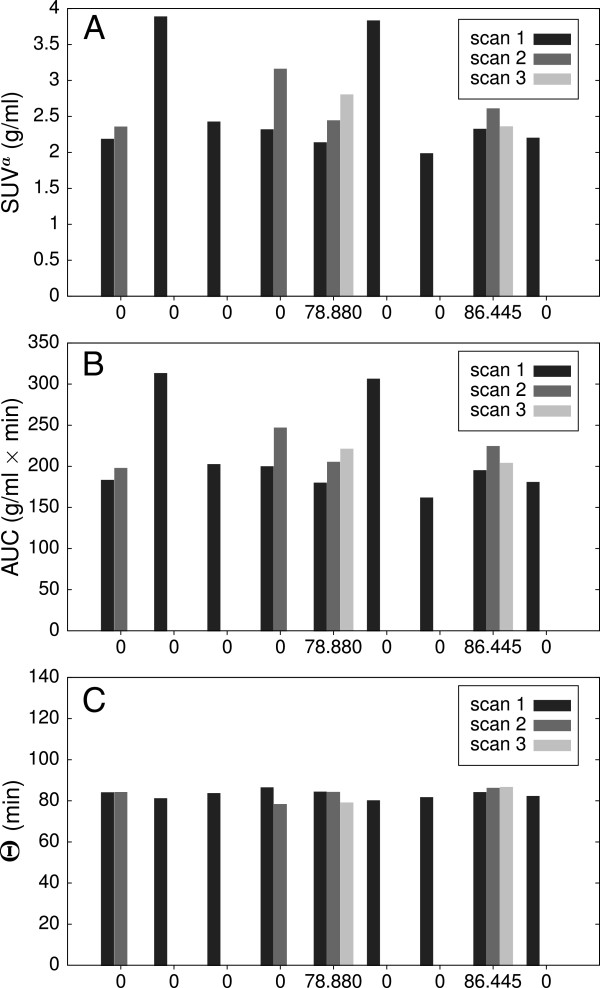
**Inter-study variability of *c***_***a***_** and of the corresponding AUC. (A)** Using SUV units in nine subjects and 15 scans and **(B)** the corresponding AUC as well as **(C)***Θ*=AUC/*c*_*a*_ at 55 min p.i.

Figure 3 compares the variability of *c*_*a*_ at *T*=55 min with that of AUC and *Θ*. Variability of AUC closely follows that of *c*_*a*_ (which can be understood as a direct consequence of approximate shape invariance of *c*_*a*_ across investigations) and amounts to a standard deviation of 20%. Due to the highly correlated variations of *c*_*a*_ and AUC, *Θ*=AUC/*c*_*a*_ exhibits a much smaller variability with a standard deviation of only 3.2% which demonstrates that *Θ* actually is approximately constant across all conducted investigations.

**Figure 4:**
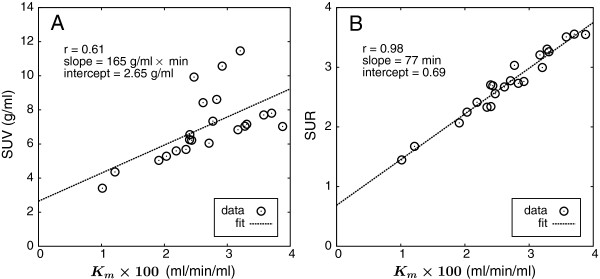
**Correlation between *K***_***m***_** and, respectively, lesion SUV (A) and SUR (B) determined at 55 min p.i.** Dashed lines represent the least-squares straight-line fits to the data.

Figure 4 shows the correlation between *K*_*m*_ and, respectively, SUV (Figure 4A) and SUR (Figure 4B) together with straight-line fits to the data.

There is only a rather weak correlation between *K*_*m*_ and SUV (*r*=0.61) while *K*_*m*_ and SUR are strongly correlated (*r*=0.98). The axis intercept of the straight-line fit in the latter case is *a*=0.69±0.10 which compares reasonably well with the average apparent volume of distribution in this patient group (as a result of the Patlak analysis) of 0.53±0.11. The slope is *m*=76.8±3.7 min which, too, is in accord with the theoretical expectation *m*≈*Θ* and with Figure 3C from which an average of 82.9±2.6 min follows for *Θ*.

## Discussion

We could demonstrate a very high linear correlation between SUR and *K*_*m*_ in the investigated patient group which is in marked contrast to the rather poor linear correlation between SUV and *K*_*m*_. In fact, the latter correlation is somewhat inferior to results reported in the literature (e.g., [5,19]) which might be traced back to the occurrence of several unusual high *c*_*a*_ values corresponding to the data points exhibiting the largest deviation from the regression line. This finding might hint at unidentified problems such as erroneous scanner calibration at the time of the respective scans although a retrospective investigation did not reveal any proof for this. Incidentally, this also demonstrates the insensitivity of SUR to such possible sources of errors. However, even when taking the literature values as reference instead of our own results, it is still true that the degree of linear correlation between SUR and *K*_*m*_ is distinctly higher than is the case when using SUV.

In theoretical terms, the linear SUR vs. *K*_*m*_ correlation can be understood as a consequence of a common input function shape and small variability of the apparent volume of distribution, *V*_*r*_. The latter was already demonstrated and discussed in [9].

The former requirement, namely the approximate shape invariance of FDG input curves after bolus injection, on the other hand, is well known to be reasonably well fulfilled. This has already been utilized in the past, e.g., in the context of population-averaged input curves ([5,7]). For completeness, it might also be noted that, in fact, all shape variations leaving the integral $\int_{0}^{T}b_{a}(s)\text{ds}$ unchanged do not influence the SUR vs. *K*_*m*_ correlation. Strictly speaking, it is not really the shape invariance of the input function that has to be assumed but only the invariance of the area under the normalized input curve *b*_*a*_(*t*) up to time *t*=*T*. Notably, this implies that the frequently observed inter-study variability of the AIF during the early (bolus passage) phase has essentially no influence since it does not contribute much to the integral up to *T* (see Figure 1). Overall, the actually observed deviations of SUR from the regression line in Figure 4B directly reflect the (quite small) cumulative effect of both *V*_*r*_ and input function shape variability in this patient group.

Contrary to SUV determination, the SUR approach requires delineation of a second ROI to derive the AIF concentration. This could be seen as an additional source of inter-observer variability and thus constitute a potential disadvantage of the approach in comparison with the SUV usage. While this is true in principle, determination of the arterial concentration does actually contribute only modestly to the observed variability of the SUR vs. *K*_*m*_ correlation (which is quite small, anyway). This was verified by repeated, intentionally differing delineations of the corresponding 3D ROI. As long as the prescription to use a substantial safety margin in the transaxial plane and a reasonable large ROI volume (extending the ROI over a sufficiently large axial section of the aorta) is respected, the reproducibility of the results is very high.

As is well known, target to reference tissue ratios have been frequently used successfully in SPECT as well as in PET (and still are), e.g. in neuro-receptor imaging, but also in oncology. In view of this fact, it is somewhat surprising that seemingly, SUR has up to now not been investigated systematically as an alternative to SUV in oncological PET. We were only able to locate a single study explicitly comparing prognostic parameters derived from tumor-to-blood ratios and SUVs, respectively, in patients with non-small cell lung cancer but without comparing these parameters to *K*_*m*_[20]. The authors reported superiority of tumor-to-blood ratios over SUV in this study without further investigating the reason for this finding. Otherwise, there is only a single methodological work we know of, namely that by Hunter et al. [5], which proposes a strategy that bears some resemblance to the SUR approach. These authors used a control-group averaged input function (modeled as a sum of three exponentials) plus single venous sampling to fix the unknown amplitudes of the exponentials. By far, the most important contribution to the time integral over the input function stems from the slowest exponential, and the described procedure therefore essentially reduces to using an invariant-shaped input function scaled to the actually observed individual late tracer concentration in the blood. The scaled input function is then integrated analytically (using the sum of exponential models) and a formula for *K*_*m*_ (called *P*_2_ in that paper) derived that is essentially equivalent to our Equation 2. Finally, these authors propose to use *c*_*t*_/AUC for *K*_*m*_ estimation, thus neglecting the influence of the term proportional to *V*_*r*_ in Equation 4 completely. Hunter et al. were nevertheless able to demonstrate that the resulting *K*_*m*_ estimate correlates highly with true *K*_*m*_ (*r*=0.98 and *r*=0.97) while SUV (called DUR in that paper) is found to exhibit a rather unsatisfactory correlation to *K*_*m*_ (*r*=0.73 and *r*=0.84). These findings are in full accord with those of the present investigation. The main difference to our work is that Hunter et al. impose the requirement of a suitable control group averaged and analytically modeled input curve plus a single blood sample in order to estimate the individual area under the input curve. This knowledge is necessary in order to estimate *K*_*m*_ in their approach. However, the implied assumption is that *Θ*=AUC/ *c*_*a*_ is constant across investigations so that, due to the neglect of the small *V*_*r*_-dependent term, SUR becomes approximately proportional to *K*_*m*_. While these conclusions are implied in the work of Hunter et al., they are not explicitly stated or utilized in their work. The present work, on the other hand, demonstrates that there is no need for either a population-averaged AIF (and assumption of a specific shape of the individual AIFs) or blood sampling in order to derive a quantity (SUR) that is linearly correlated to *K*_*m*_. We believe this very distinct linear correlation to be the practically most relevant aspect. Whether conversion from SUR to *K*_*m*_ does always make sense is not so obvious as will be discussed below.

Compared to tissue SUV, the use of SUR eliminates a couple of shortcomings of the former. For one, SUR is a dimensionless quantity that is neither affected by inaccuracies of scanner or dose calibration nor by erroneous body weight (or related quantities). While this is clearly advantageous, the most relevant improvement of SUR over SUV in our view is elimination of a very large part of the variability caused by inter-scan variations of the input function. Given the empirically well demonstrated approximately invariant shape of FDG input functions, SUR eliminates the influence of inter-scan variations of tracer supply nearly completely. Quite to the contrary, variations in the scale of *c*_*a*_(*t*) (if measured in SUV units) directly translate into substantial SUV changes. As Figure 2 demonstrates, the inter-subject as well as the intra-subject variability of *c*_*a*_ is indeed substantial (exhibiting a standard deviation of about 23% from the mean) which directly explains the large fluctuations of the SUVs observed in Figure 4A. It should be stressed, that this variability of *c*_*a*_ persists *after* conversion to SUV units (or to SUV_lbm_ or SUV_bsa_, for that matter). In other words, the central objective of SUV-based approaches, namely correction for variations of injected dose and systemic distribution volume of the tracer, is not achieved satisfactorily. Consequently, comparing SUV values across investigations makes only limited sense and is prone to potentially serious errors if the objective is to assess changes of glucose metabolism. Inter-study comparison of SUR values appears much more reasonable in this respect. At least since the advent of PET/CT a purely image-based determination of the blood signal from a large vessel is straightforward even where this would not be quite as easy when using the whole body PET data alone. Therefore, SUR computation does especially not require any blood sampling and could also easily be performed retrospectively using existing data for systematic performance comparisons of SUV and SUR.

As demonstrated, replacing SUVs by SURs does eliminate some important sources of SUV-specific systematic errors which leads to a very good linear correlation between SUR and *K*_*m*_. On the other hand, this does not imply that SURs are necessarily also suitable for quantitative determination of *K*_*m*_ in general since the SUR vs. *K*_*m*_ correlation shown in Figure 4B need not be universally valid for two reasons. First, according to Equation 3, the slope of the SUR (*K*_*m*_) correlation is identical to the integral over *b*_*a*_(*t*) up to *t*=*T*. Different injection protocols (duration of injection, bolus vs. ramp etc.) naturally will thus lead to a different (and unknown) slope parameter. However, even if the invariant shape assumption can be considered universally valid, the slope remains a function of *T* and thus will change (in an unknown way) if the time of measurement is changed.

Second, the intercept of the regression line approximates the average apparent volume of distribution, ${\bar{V}}_{r}$, and thus might vary across different tumor entities and tissues. We believe this probably to be a small effect for most tumors and healthy tissues (although this cannot be taken for granted and would need to be proven by further investigations), but for certain tissues and when inflammation is involved (i.e., if the assumption of irreversible trapping is not fulfilled), larger variations of *V*_*r*_ will occur, and consequently, the relation between SUR and *K*_*m*_ will be distinctly modified. Obviously, the presence of a sizable dephosphorylation (i.e., a sizable *k*_4_ in the corresponding two-compartment model) would actually invalidate the prediction of a linear SUR vs. *K*_*m*_ correlation completely. It should be noted, however, that this is not a specific problem of the SUR approach but affects SUV evaluations accordingly. In the present study, inspection of the Patlak plots unambiguously demonstrated that the FDG kinetics in the liver lesions is compatible with the assumption *k*_4_=0.

Still, in general, it cannot be expected that the regression line in Figure 4B is universally valid (at least not with good accuracy), and quantitative conversion of SUR to *K*_*m*_ should probably be avoided in our view (and would not offer additional benefits).

Instead, we surmise that it might be worthwhile to combine the SUR approach with a dual time point-based determination of *K*_*m*_[9] in order to identify regions in which the SUR vs. *K*_*m*_ relation is changed. This possibly could, e.g., enable a more sensitive identification of inflammatory processes than is possible with dual time point measurements alone.

Where such dual time point protocols allowing for independent determination of *K*_*m*_ and SUR are considered not compatible with the requirements of clinical routine, SUR alone still offers distinct advantages over SUV-based approaches because a good linear correlation to *K*_*m*_ similar to that shown in Figure 4B can be expected in most tissues and target structures (i.e., those where FDG kinetics can be considered to be irreversible): a fractional change of SUR will in general correspond to a concomitant change of *K*_*m*_ where even modest SUR changes will be meaningful due to the very high degree of linear correlation of both quantities (as long as a sufficiently accurate determination of *c*_*a*_ within the image data is performed that avoids partial volume and spill over effects). Inter-study SUV differences, instead, are likely to be partially or even completely spurious (i.e., unrelated to *K*_*m*_ changes) due to the insufficient correction for inter-study variability of arterial tracer concentration achieved by the SUV approach.

## Conclusions

The tumor-to-blood standard uptake ratio, SUR exhibits a much higher linear correlation to the metabolic rate of FDG, *K*_*m*_, than tumor SUV (or SUV_lbm_ or SUV_bsa_). This improved correlation can be understood theoretically as a consequence of the approximate shape invariance of FDG input functions. Purely image-based determination of SUR is straightforward in whole body investigations and allows to account for variations in tracer supply across investigations distinctly better than when using SUVs. Therefore, SUR should be seriously considered as a drop-in replacement for SUV-based approaches.

## Competing interests

The authors declare that they have no competing interests.

## Authors’ contributions

JVDH had the initial idea for SUR, performed part of the data analysis, and wrote part of the manuscript. LO and GS performed part of the data analysis. JM, AL, and JP provided intellectual input and reviewed the manuscript. BBB selected the patient studies and performed the lesion delineation. FH performed part of the data analysis and wrote part of the manuscript. All authors read and approved the final manuscript.
